# Distributed transmission power control for communication congestion control and awareness enhancement in VANETs

**DOI:** 10.1371/journal.pone.0203261

**Published:** 2018-09-05

**Authors:** Hyeongjun Chang, Young Eun Song, Hyungoo Kim, Hoeryong Jung

**Affiliations:** 1 Department of Mechanical Engineering, Konkuk University, Seoul, Korea; 2 Department of Electrical Engineering, Hoseo University, Cheonan, Korea; 3 Department of Mechanical Engineering, Korea Advanced Institute of Science and Technology, Daejeon, Korea; Universita degli Studi della Tuscia, ITALY

## Abstract

The vehicular ad hoc network (VANET) has been identified as one of the most promising technologies for managing future intelligent transportation systems. This paper proposes a distributed transmission power adjustment algorithm for communication congestion control and awareness enhancement to address communication congestion problems that can arise in VANETs. The objective of the proposed algorithm is to provide maximum awareness of surrounding vehicles’ status while maintaining a communications channel load below the allowed threshold. The proposed algorithm accomplishes this by adjusting the transmission range of each vehicle in the network progressively and gradually, while monitoring the communications channel load of each vehicle. By changing the transmission range of a vehicle little by little according to the communications channel load of its neighboring vehicles, the algorithm finds the optimal transmission range that provides maximum awareness without resulting in communications congestion. In addition, the proposed algorithm appropriately controls the channel load in a fair manner without sacrificing awareness of specific vehicles in the congested situation. This allows nearby vehicles to obtain more peripheral information to help them stay away from potential hazards and maintain safety. The proposed algorithm was implemented in a simulation environment, and its performance was validated in various traffic scenarios. The simulation results show that the proposed algorithm can deal with communication congestion by controlling the transmission power fairly to a target threshold in various traffic situations.

## Introduction

One of the central features of modern cities across the world is the large concentration of population within a relatively small area. Additionally, as cities in many countries continue to grow, this density increases as well. Consequently, the number of cars, and the concurrent and conflicting necessity for the movement and transport of people and products, also increases. This results in heavy traffic congestion almost every day in many urban areas, causing an increase in time losses for traffic participants, an increase in environmental pollution and noise, and an increase in the number of traffic accidents. According to the U.S. National Highway Traffic Safety Administration, in 2015, 35,092 people died, and 2,440,000 people were injured in motor vehicle traffic crashes in the United States alone [1]. It is safe to say that these statistics are not unique to the United States; a large number of people are killed or injured in traffic accidents every year in many other countries.

Several initiatives [2] have been launched with the objective of decreasing both the number of accidents and their resulting damage. These efforts not only attempt to improve traffic handling and road systems, they also introduce innovative technologies that enhance the drivers’ awareness of the surrounding traffic situation. Among the recent technologies proposed to improve road safety, vehicular ad hoc networks (VANETs) are quite promising, because their use of wireless communications offers the beneficial capability of directly exchanging safety-related information between vehicles. In VANETs, each vehicle wirelessly shares its driving information (such as position, direction, and speed) with neighboring vehicles, so that all vehicles have an accurate image of traffic conditions on the road. This has the advantage of providing accurate driving information about neighboring vehicles regardless of the presence of line-of-sight obstacles. Recently, VANET research groups have been developing technology combining wireless communications (on the IEEE 802.11 standard) with on-board sensors (e.g., GPS, speedometers) in order to improve the driver’s awareness of the surrounding environment.

VANETs are based on standard wireless LAN technologies, such as IEEE 802.11p, but differ in some significant ways from existing WLANs. Because VANETs are intended for use in vehicles, the traditional handshaking method commonly used in WLANs is difficult to apply, in part because of the highly variable network topology. Therefore, a one-hop broadcasting method has been employed for the exchange of messages in VANETs instead of the traditional handshaking method. However, if a one-hop broadcasting technique is used, a communications channel can become overloaded in a crowded environment and communications congestion may occur, because of the nature of information exchanged in the VANET broadcast channel, which includes not only event-driven messages that occur during an emergency, but also periodic messages (referred to as beacons in this paper) that indicate the current status of the vehicle, such as location, speed, and direction. As the number of vehicles in a relatively small area increases, the communications channel becomes overwhelmed by beacon information, and event-driven messages can be lost or delayed in the congestion.

Since the establishment of the WAVE standard in 2010 [3], much research has been done on ITS applications in VANETs, but research on safety applications is still an active and important area. In particular, the performance of beacons in vehicle-to-vehicle (V2V) communication for cooperative safety applications is being studied as an important element in supporting high-level applications. In the United States, beacons based on periodic broadcasts are defined as basic safety messages (BSMs) [4], and basic congestion control solutions, including transmit rate and power control, were presented in the standard document (SAE J2945/1) [5]. This standard provides the transmit rate control algorithm, which determines the maximum inter-transmit time between two consecutive BSMs depending on the vehicle density. It also presents the transmit power control method, which determines the transmit power depending on channel busy percentage (CBM). The European Telecommunications Standards Institute (ETSI) defined beacons as cooperative awareness messages (CAM) and established a framework for decentralized congestion control (DCC) including transmit power, rate, and message rate in ETSI TS 102 687 [6].

The congestion control performance is closely related to safety application performance. It is ideal to have a short beacon period, a high reception ratio, a short delay time, and a wide transmission radius. However, as the beacon period shortens and the transmission radius widens, the beacon collision probability increases and the beacon reception rate decreases. Therefore, most studies to improve beacon performance have focused on selecting optimal parameters and determining the values considering vehicle condition and network environment. Typical control methods for determining the performance of a beacon include beaconing rate control, transmit power control, and data rate control [7]. The core technology of these control techniques is to find optimized parameter values for a given situation. Though it may seem desirable to reduce the size of the beacon message or its transmission period in order to reduce beaconing load in congested situations, these can lead to dangerous situation because a lack of information can threaten the safety of the vehicles in the system. The best solution, then, for the reduction of beaconing load in a congested situation is to reduce the number of vehicles exchanging data with each other. This reduction in communications congestion is practically implemented by reducing the transmission range of each vehicle, which is accomplished by adjusting the signal transmission strength.

This paper proposes a distributed power control algorithm that controls the signal transmission range of the vehicles in a VANET to prevent communications congestion in a crowed traffic situation. This algorithm ensures the effective exchange of as much information as possible between vehicles by providing the optimal usage of beaconing channel load without violating the assigned emergency channel load. Because it is based on the relative distance between vehicles, the algorithm provides an efficient transmission power control strategy without generating an increase in communications overhead. The authors present the following contributions:

- An algorithm is proposed that ensures the beaconing load of each vehicle does not exceed the maximum allowed beaconing load by optimally controlling the transmission range of each vehicle.
- The proposed algorithm maximizes the transmission range of each vehicle without causing communication congestion. Thus, the maximum possible amount of information regarding the surrounding vehicles is available to each vehicle fairly in the VANET.

The proposed algorithm can deal with communication congestion by controlling the transmission power close to a maximum allowed beaconing load. The performance of the proposed algorithm was evaluated through simulations of various traffic scenarios.

### Related work

Numerous methods have been proposed to solve the channel congestion problem in VANETs, and those can be classified into three types: beaconing rate control, transmit power control, and hybrid control of transmission power and rate. This section reviews these three previous types of congestion control methods.

#### Beaconing rate control

Transmit rate control (TRC), which controls the beaconing period, is the technique that has the greatest effect on the beacon reception rate. The adaptive traffic beacon (ATB) proposed by Sommer [8] is a representative method of the TRC scheme that determines the beacon transmission period based on the channel quality and the message utility. Kim [9] proposed a distributed but coordinated control scheme to solve the problem of a threshold- or hysteresis-based control scheme. They demonstrated that the control method provably leads to stability and relevance to the given vehicle density pattern. Egea-Lopez [10] proposed a model for the network utility maximization (NUM) problem to adequately control the trade-off between efficiency and fairness. They also proposed a FABRIC algorithm using a particular scaled gradient projection algorithm to solve this problem. In a subsequent study [11], the authors also pointed out that previous studies have focused on fairness rather than efficiency in studying beacon control, which may be problematic for vehicle safety. To solve this problem, the authors proposed to let vehicles transmit with different transmit power levels, each with a particular beaconing rate. As a result, the number of beacons delivered at each transmit power is maximized according to a well-defined fairness notion while complying with the maximum allowed beaconing load on the channel.

#### Transmission power control

Beaconing rate control can lead to a potential lack of information, which can threaten the safety of the vehicle because it reduces the beaconing rate in a congested situation. On the other hand, the transmit power control method, which adjusts the communication range of each vehicle by controlling its beacon transmit power, can stably provide the driving status of nearby vehicles. Artimy [12, 13] proposed a model to predict the optimal range for maximizing one-hop broadcast coverage using information about the network density and node sending rate. An initial attempt at considering topology control for VANETs was proposed, in which transmission ranges are dynamically adapted to the rapidly changing vehicular topology. Unfortunately, this network connectivity-based transmit power control strategy is insufficient for VANETs’ safety-related objectives. Torrent-Moreno [14] presented a fair bandwidth sharing approach for VANETs. This approach limits the wireless load resulting from the periodic beacon messages by implementing strict fairness among vehicles. Accordingly, this approach is called the fair power adjustment for vehicular environment (FPAV) algorithm. Conceptually, vehicles using the FPAV algorithm adjust their transmit power using power control techniques in such a way that the bandwidth utilized by periodic beacon messaging does not exceed a predefined threshold known as the maximum beaconing load (MBL). The idea behind defining the MBL is to reserve a fixed quantity of bandwidth for event-driven messages (in emergency situations) so that the communication of safety applications is not hindered by a channel saturated with beacon information. Considering the drawbacks of the FPAV algorithm, the same researchers designed an enhanced and fully distributed version of this algorithm called the distributed fair power adjustment for vehicular networks (D-FPAV) algorithm in [15, 16]. The D-FPAV algorithm dynamically adjusts each vehicle’s transmission power (and hence transmission range) to prevent packet collisions. This algorithm was formally proven to follow the max–min fairness criterion. However, because the one-hop broadcast of beacon messages is insufficient to acquire the beacon load of neighboring vehicles within the communication range, it is necessary to transmit additional information about neighbor status. Because of this, the authors of the D-FPAV algorithm used extended beacon messages piggybacked onto every n beacon messages and containing a list of the neighboring vehicle status information. Unfortunately, this extended beacon message also increases the beaconing load in the control channel. This indicates the presence of a tradeoff between accuracy and overhead in VANETs. Mittag [17] presented an analysis of the D-FPAV algorithm, including the tradeoff between the increased overhead caused by beacon information exchange and the accuracy of the status information of the surrounding nodes. To reduce the communications overhead generated by the D-FPAV algorithm, Mittag introduced the distributed vehicle density estimation (DVDE) and the segment-based power adjustment for vehicular environments (SPAV) strategies. Lu [18] proposed a novel transmit power control protocol that aims to improve the reliability of safety-critical broadcasts while also maximizing their transmission range. A balance was required between maximizing the number of packets successfully received by nearby vehicles and maximizing the transmit power to reach vehicles at greater distances. Note that this proposed protocol incurred minimum additional overhead. A wireless access in vehicular environments (WAVE) short message protocol was proposed in [19], which made use of a Markov-chain model to compute the dropping probability and expected delay of safety-related packets for mitigating the congestion problem. Though they provided a discard-based congestion control scheme at road site units, this approach may not be acceptable in a VANET environment because of the dynamic nature of the network. Mo [20] proposed a transmit power control algorithm based on channel load forecasting. The author was able to control the channel congestion within a predetermined range by pre-adjusting the transmission power through the CLF-BTPC algorithm after predicting the channel load using the KF-BCLF algorithm based on the recursive Kalman filter.

#### Hybrid control of transmit power and rate

Huang [21] proposed a transmit rate and power control function to obtain acceptable accuracy for tracking neighboring vehicles while avoiding congestion in a shared communications channel. The power control used is a linear function set between the minimum and maximum power constraints of the channel load, as measured by the clear channel assessment function. Transmit events are chosen statistically as a function of the clear channel assessment, the perceived packet error ratio, and vehicle dynamics, in an attempt to control a receiver’s modeling error and to use the channel efficiently. Baldessari [22] proposed an approach that controls the combination transmit power and rate based on the information of the neighboring nodes and concluded that the combination results in better performance than separate algorithms and reduces the power control requirements on front-ends by leveraging on packet rate control. Tielert [23] proposed joint power and rate control algorithms to achieve congestion control in vehicle safety communications. They concluded that an efficient strategy is to select transmit power according to the target awareness distance and to adapt the message rate according to the channel load.

## Materials and methods

From the problem description outlined in the previous section, we propose the distributed transmission power control algorithm for communication congestion control and awareness enhancement in VANETs. The proposed algorithm can be used in VANETs in the following manner:

*WHEN*? If the beaconing load (*BL*) exceeds the maximum allowed beaconing load (*MABL*), it is time to control the transmission power. A vehicle (*node*) whose *BL* exceeds the *MABL* is referred to as a *distressed node*.

*WHO*? If a node is in distress, its neighboring nodes, who are transmitting messages to the *distressed node*, then adjust their transmission power.

*HOW*? A *distressed node* broadcasts a distress message to the nodes in its maximum communication range. The nodes that receive the distress message reduce their communication range gradually by a predefined decrement until the distressed situation is completely resolved.

### Parameter definition

We defined several parameters to describe the proposed algorithm, assuming that a set of nodes ***N*** = {*u*_1_,⋯*u*_*n*_} is moving along a road ***R*** = [0,1], which is defined as a line.

Definition 1) Power Assignment:

Given a set of nodes ***N*** = {*u*_1_,⋯*u*_*n*_} a power assignment ***PA*** = {*PA*_1_,⋯*PA*_*n*_} is defined as a set of scalar values that assigns transmission power to the node *u*_*i*_ ∈ ***N***. The value of *PA*_*i*_ is defined as a ratio between 0 and 1 at $P_{i}^{max}$, which represents the maximum transmission power of the node *u*_*i*_. The transmission power of the node *u*_*i*_ is then calculated by ${{PA}_{i}∙P}_{i}^{max}$.

Definition 2) Communication Range:

The communication range of node *u*_*i*_ under *PA*_*i*_, which is denoted by *CR*_*i*_, is defined as the distance up to which a node’s message can be successfully received by another node. The communication range of the node *u*_*i*_ at maximum transmission power is denoted as ${CR}_{i}^{max}$.

Definition 3) Neighbor Nodes:

The neighbor nodes of the node *u*_*i*_, which are denoted as ***NEI***_*i*_, are defined as the set of nodes that transmit messages to the node *u*_*i*_:

$${NEI}_{i}=\{u_{j}|u_{j}\in N,j\neq i\;and\|u_{i}-u_{j}\|<{CR}_{j}\}$$

Definition 4) Beaconing Load:

The beaconing load of node *u*_*i*_ is defined as the amount of beaconing data transmitted to it from its neighbor nodes:

$${BL}_{i}=\sum_{u_{j}\in {NEI}_{i}}{\left(Beacon\;size\right)}_{j}\times {\left(Transmission\;rate\right)}_{i}\;[\mathrm{bytes}/\sec]$$

*Definition 5) Per-node Maximum Beaconing Transmit Power Problem* (*PMBTxP*(*u*_*i*_)):

Determining a maximum value of *PA*_*i*_ under the constraints that *BL*_*j*_ (the beaconing load of *u*_*j*_ ∈ ***NEI***_*i*_) remains below the *MABL*:

$$Maximize\;PA_{i}$$

subjectto

$$BL_{j}\leq MABL\;where\;u_{j}\in NEI_{i}$$

### Distributed power control algorithm

The proposed distributed power control algorithm is described in Table 1. This algorithm is referred to as Algorithm 1 in the following text.

**Table 1:** Distributed transmission power control algorithm.

Algorithm 1: Distributed transmission power control for node *u*_*i*_	
Input:	Beaconing messages from *u*_*i*_’s neighbor nodes *u*_*j*_ ∈ *NEI*_*i*_
Output:	A power assignment *PA*_*i*_ that satisfies *PMBTxP*(*u*_*i*_)
1:	**if** *BL*_*i*_ ≤ *MABL* **then**
2:	**if** *DISTRESS*_*i*_ *= true* **then**
3:	*PA*_*i*_ = *max*(*PA*_*i*_ − *ε*,*PA*_*min*_)
4:	**else**
5:	*PA*_*i*_ = *min*(*PA*_*i*_ + *ε*,*PA*_*max*_)
6:	**endif**
7:	**else**
8:	Broadcast ‘distress’
9:	**endif**

**Fig 1:**
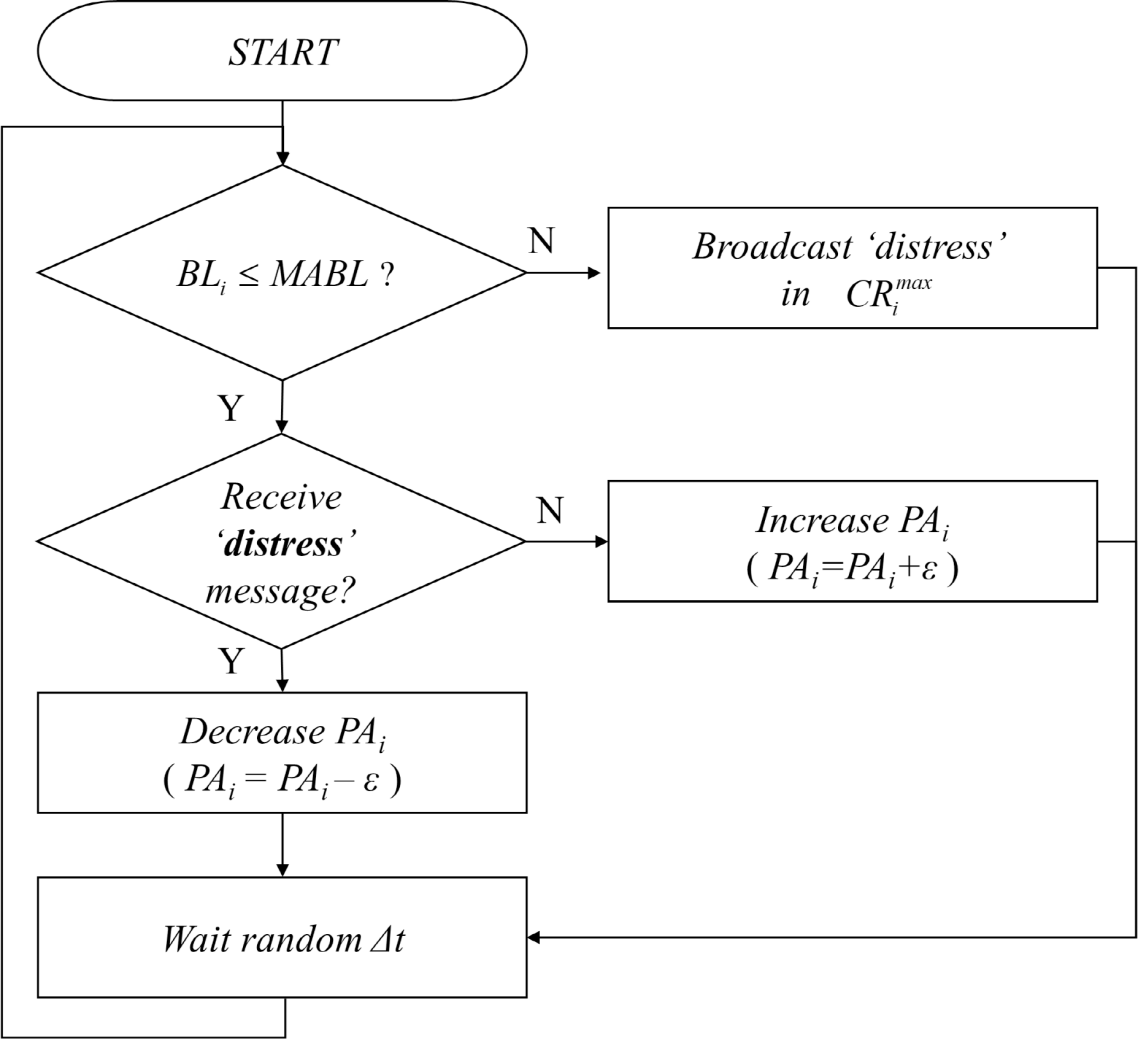
Flow chart of the distributed transmission power control algorithm.

All nodes periodically transmit beacon messages (containing data such as position, speed, velocity, and distress) to the nodes in their communication range *CR*_*i*_. At initiation, the algorithm first checks whether its beaconing load *BL*_*i*_ exceeds *MABL*. If *BL*_*i*_ exceeds *MABL*, the node *u*_*i*_ broadcasts a distress message to the neighbor nodes that lie within ${CR}_{i}^{max}$, and waits a random interval of time Δ*t*. If *BL*_*i*_ does not exceed *MABL*, then the node *u*_*i*_ checks whether or not it has received a distress message. If the node *u*_*i*_ has not received a distress message, then it increases its power assignment *PA*_*i*_ by *ε* and waits Δ*t*. If the node *u*_*i*_ receives a distress message, then it decreases its power assignment *PA*_*i*_ by *ε* and waits Δ*t*. The flow chart of the Algorithm 1 is illustrated in Fig 1.

### Algorithm verification

**Fig 2:**
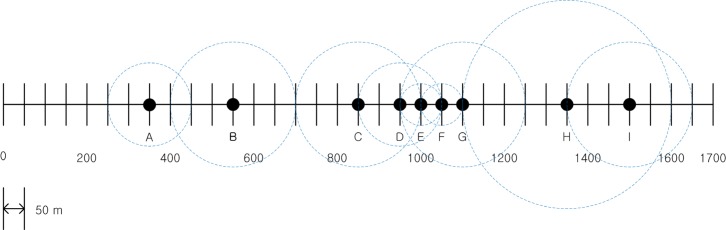
Initial node deployment and *CR* of nodes in the verification scenario.

The simple traffic scenario presented in Fig 2 was used to verify the validity of the proposed algorithm. In this scenario, there are nine vehicles, denoted as *A*,⋯,*I*, placed along a 1.7-km-long road segment, with relative distances varying from 50 m to 300 m. The communication range of each vehicle can be varied by multiples of a unit distance of 50 m. In this scenario, we assumed the following VANET communication conditions:

- The relative positions of all nodes are fixed, and only the communication range of each node varies, according to the proposed algorithm.
- *CR*^*max*^ of each node is set to 400 m and the minimum *CR* is set to 50 m
- If *PA*_*i*_ is increased or decreased by *ε*, then *CR*_*i*_ is increased or decreased by 50 m accordingly.
- The packet size and transmission rate is set to one, such that *BL*_*i*_ = *n*(***NEI***_*i*_)
- In each time step, only one node can increase its power assignment.
- The value of *MABL* is set to 4.

Initially, the *CR* of each node was set randomly as shown in Fig 2. Each node then began to increase its *PA* by *ε*, so that its *CR* increased by 50 m in each step. This increasing procedure continued for one node per step until a node broadcast a distress message. When a node broadcast the distress message, the neighbor nodes that received the distress message decreased their *PA* by *ε* so that their *CR* decreased by 50 m. The scenario proceeded in this manner for 12 steps. The verification results are shown in Table 2, in which the numbers represent the beaconing load of each node. The node that increased its *CR* was selected randomly at each step, and the names of the selected nodes are presented in the leftmost column in the table.

**Table 2:** Step-by-step summarization of the algorithm execution (with *MABL* = 4).

Selected Node	Step	A	B	C	D	E	F	G	H	I
-	Initial BL	0	0	1	3	4	3	2	1	1
B	1st step BL	1	0	1	3	4	3	2	1	1
I	2nd step BL	1	0	1	3	4	3	2	1	1
C	3rd step BL	1	0	1	3	4	4	2	1	1
F	4th step BL	1	0	1	4	4	4	2	1	1
H	5th step BL	1	0	1	4	4	5	2	1	1
C,D,E,G,H	6th step BL	1	0	0	3	4	2	2	1	1
G	7th step BL	1	0	0	4	4	2	2	1	1
D	8th step BL	1	0	1	4	4	3	2	1	1
H	9th step BL	1	0	1	4	4	4	2	1	1
E	10th step BL	1	0	1	4	4	4	3	1	1
A	11th step BL	1	0	1	4	4	4	3	1	1
D	12th step BL	1	2	1	4	4	4	4	1	1

**Fig 3:**
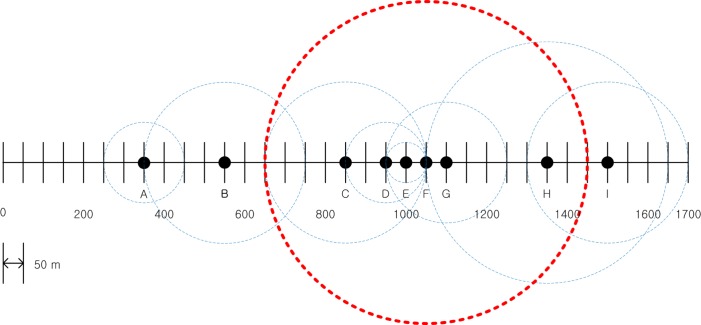
*CR* of nodes when the node (*F*) transmits the distress message.

**Fig 4:**
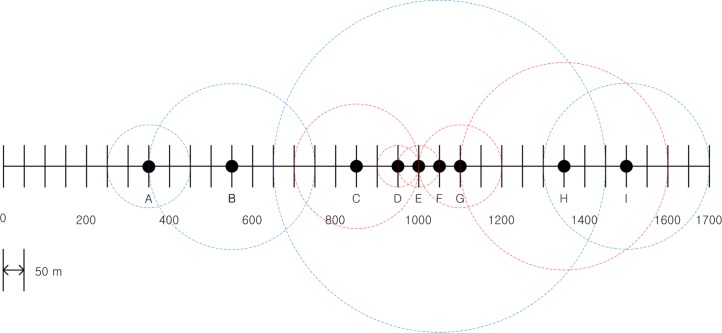
Transmission range reduction in the nodes receiving the distress message.

In the fifth step, *BL*_*F*_ can be seen to first exceed the *MABL*, as shown in the table. At that time, node *F* broadcast a distress message to the nodes within ${CR}_{i}^{max}$, which is shown as the thick red dashed circle in Fig 3. All nodes that received the distress message, i.e., *C*,*D*,*E*,*G*,*H* decreased their *CR* values to reduce *BL*_*F*_ as reflected in Table 2. The decreased *CR* values of the nodes *C*,*D*,*E*,*G*,*H* are shown in Fig 4 as red dashed circles. It can be seen in Table 2 that as a result of the distributed power control algorithm, the *BL* of most nodes reaches, but is maintained below, the *MABL* for the remainder of the procedure, which indicates that the algorithm provides substantial awareness of the surrounding vehicles without causing communications congestion.

### Distributed power control algorithm with enhanced fairness

**Fig 5:**
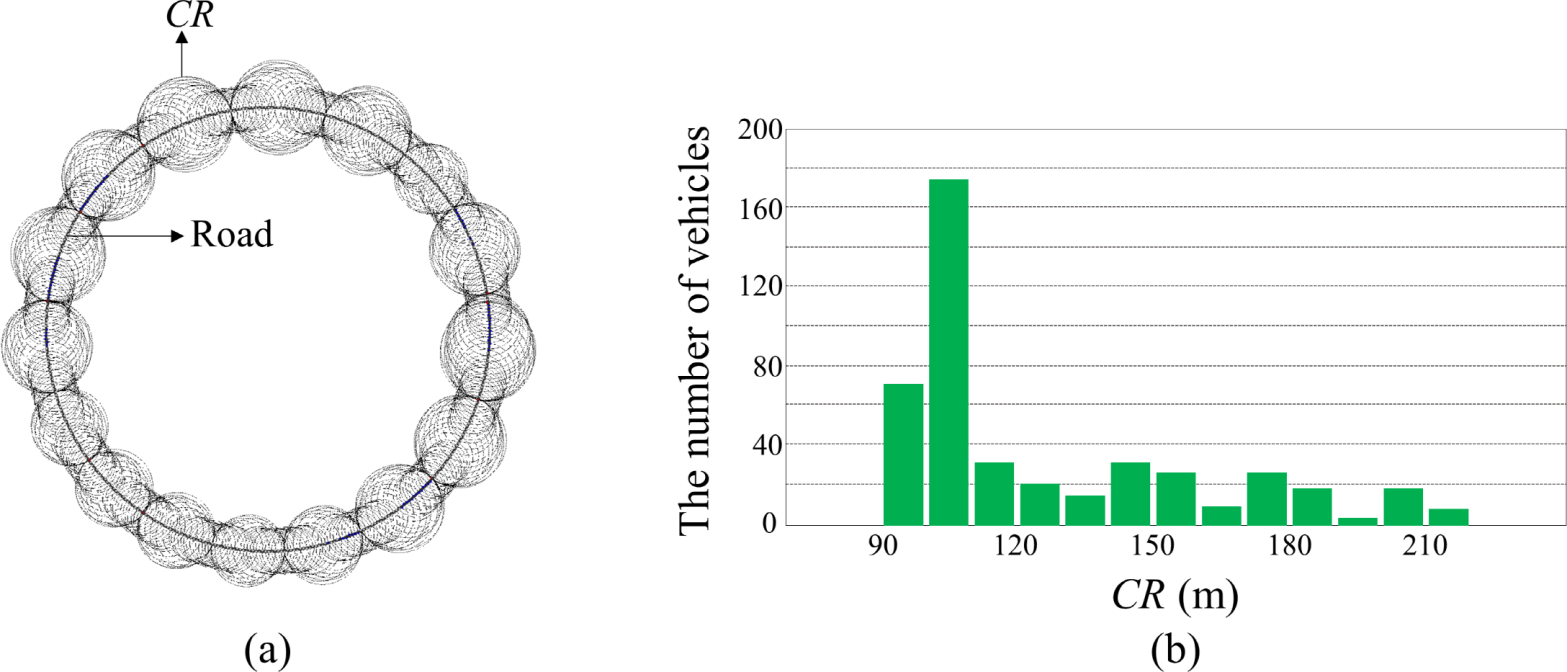
*CR* controlled by the Algorithm 1. (A) Simulated traffic situation with *CR* (B) Distribution of *CR*.

The Algorithm1 adaptively adjusts the transmit power of each node based on a pre-defined threshold value (*MABL*) to control the *BL* of the nodes in the network close to the *MABL*. The threshold-based congestion control approach has the advantage that it can efficiently utilize the network resources to maximize the vehicles’ awareness of neighboring driving status, but it presents stability and fairness issues [9]. In a real traffic situation, various noises occur during beacon transmission and reception, so a certain portion of the measured *BL* value is occupied by the noise. When the *BL* is controlled close to the *MABL*, the measured *BL* can oscillate around the threshold value due to the noise, and this leads to a severe convergence problem. Then, the distress state can be triggered with high frequency due to the noise even in static traffic situations. Fairness is also a critical issue in the threshold-based approach. The congestion control algorithm should balance two competing factors: maximizing total usage of given network resources and ensuring fairness of resource allocation. The network resources should be fairly allocated to the nodes participating in congestion control to ensure the minimum safety of the nodes. Fig 5 shows the *CR* of the nodes that are controlled using the Algorithm 1 in a simple traffic situation. In this simulation, 440 vehicles were placed equidistant on a double-lane circular race track with a 1 km radius. Since the vehicles were placed with uniform density, the controlled transmit power was expected to converge to similar values leading to similar *CR* across all vehicles. However, the resulting *CR* shown in Fig 5 deviate significantly. The *CR* values of the vehicles varied in the range between 90 and 210 m. The average *CR* was 126.31 m, and the standard deviation was 33.37 m.

**Fig 6:**
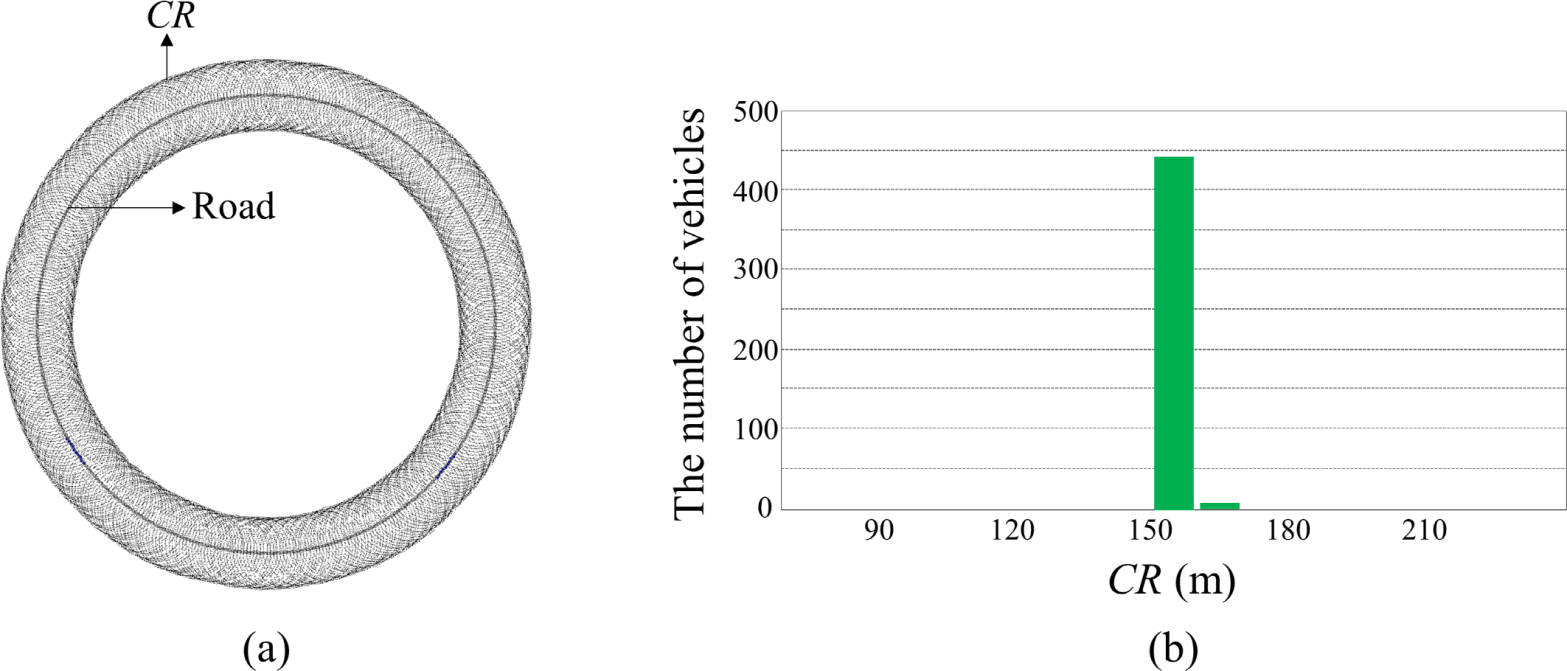
*CR* controlled by the Algorithm 2. (A) Simulated traffic situation with *CR* (B) Distribution of *CR*.

To address the stability and fairness problems of the Algorithm 1, we benchmarked the previous mean-checked rate control approach [9]. The mean-checked control algorithm compares the transmit rate of a node with the average transmission rate of its neighboring nodes when it decides to adjust the rate. The algorithm increases the transmission rate only when the current rate is smaller than the average transmission rate of its neighboring nodes. Similarly, it decreases the transmission rate only when the value is larger than the average value. Thus, the algorithm prevents an undesirable feedback cycle, which would increase deviation of the transmission rate between neighboring nodes, and accordingly maintains the transmission rate of nearby nodes at similar levels. We adopted this approach in the Algorithm 1. Table 3 shows the transmit power control algorithm with enhanced stability and fairness which is referred to as Algorithm 2 in the following text. To implement this algorithm, each node should be informed of the transmit power of its neighboring nodes. In the BSM format, the value of transmit power can be carried in Part II as optional content. Each node broadcasts its transmit power level carrying on the beaconing message to the nodes in its *CR* so that the neighboring nodes receiving the beaconing message can compute the average transmit power level of its neighboring nodes. In this algorithm, the average transmit power level of the neighboring nodes (${\bar{PA}}_{i}$) is additionally considered before increasing the transmit power (line 6). Unlike to the previous mean-check algorithm, we do not check neighboring transmit power level in the decreasing state because it can lead to network congestion. If the node receiving distress message cannot decrease its transmit power due to the neighboring power level, then the distressed state cannot be resolved, and eventually network congestion will occur. Thus, the nodes receiving distress message decrease its *CR* without considering neighboring power level to resolve distress state immediately and prevent network congestion. In this way, the Algorithm 2 maintains the deviation of *CR* among nearby nodes within an allowable range without causing network congestion. Fig 6 shows the simulation results of the Algorithm 2. The *CR* of most nodes (435 nodes among 440 nodes) were in the range of 150 to 160 m. This means that the proposed algorithm fairly assigns the transmit power to the nodes participating in congestion control.

**Table 3:** Distributed transmission power control with fairness enhancement.

Algorithm 2: Distributed transmission power control with enhanced fairness for node *u*_*i*_	
Input:	Beaconing messages with *PA*_*j*_ from *u*_*i*_’s neighbor nodes *u*_*j*_ ∈ *NEI*_*i*_
Output	Power assignment *PA*_*i*_ that satisfies *PMBTxP*(*u*_*i*_)
1:	Compute ${\bar{PA}}_{i}$
2:	**if** *BL*_*i*_ ≤ *MABL* **then**
3:	**if** *DISTRESS*_*i*_ = *true* **then**
4:	*PA*_*i*_ = *max*(*PA*_*i*_ − *ε*, *PA*_*min*_)
5:	****else****
6:	**if** ${PA}_{i}<{\bar{PA}}_{i}$ **then**
7:	*PA*_*i*_ = *min*(*PA*_*i*_ + *ε*, *PA*_*max*_)
8:	****end if****
9:	****end if****
10:	****else****
11:	Broadcast ‘distress’
12:	****end if****

## Results and discussion

### Simulation setup

The performance of the proposed distributed power control algorithm was evaluated in static and dynamic traffic situations using simulations. We used in-house simulation tool, which is implemented on a Windows 10 platform using C++, to simulate VANET communication and vehicle mobility. The proposed distributed transmit power control algorithms were implemented in the simulation, and the performance was evaluated in three different scenarios (one static and two dynamic scenarios). Table 4 shows the communication parameters of VANET used in the simulation. Each vehicle broadcast 10 beacons/s, and the size of each beacon was set to 500 bytes. The value of *MABL* was set to 2.5 Mbps. A deterministic two-ray ground model was used to simulate the attenuation effect of transmission signals present in a VANET implementation.

**Table 4:** VANET simulation parameters.

**Parameter**	**Value**
*Data rate*	3 Mbps
*Sensitivity*	-92 dBm
*Frequency*	5.9 GHz
*Noise*	-110 dBm
*MABL*	2.5 Mbps
*Beacon generation rate*	10 packets/sec
*Beacon size*	500 bytes
*Radio propagation model*	Two-ray ground
*Sample period*	1 s

### Simulation results

#### Scenario 1 (static)

**Fig 7:**
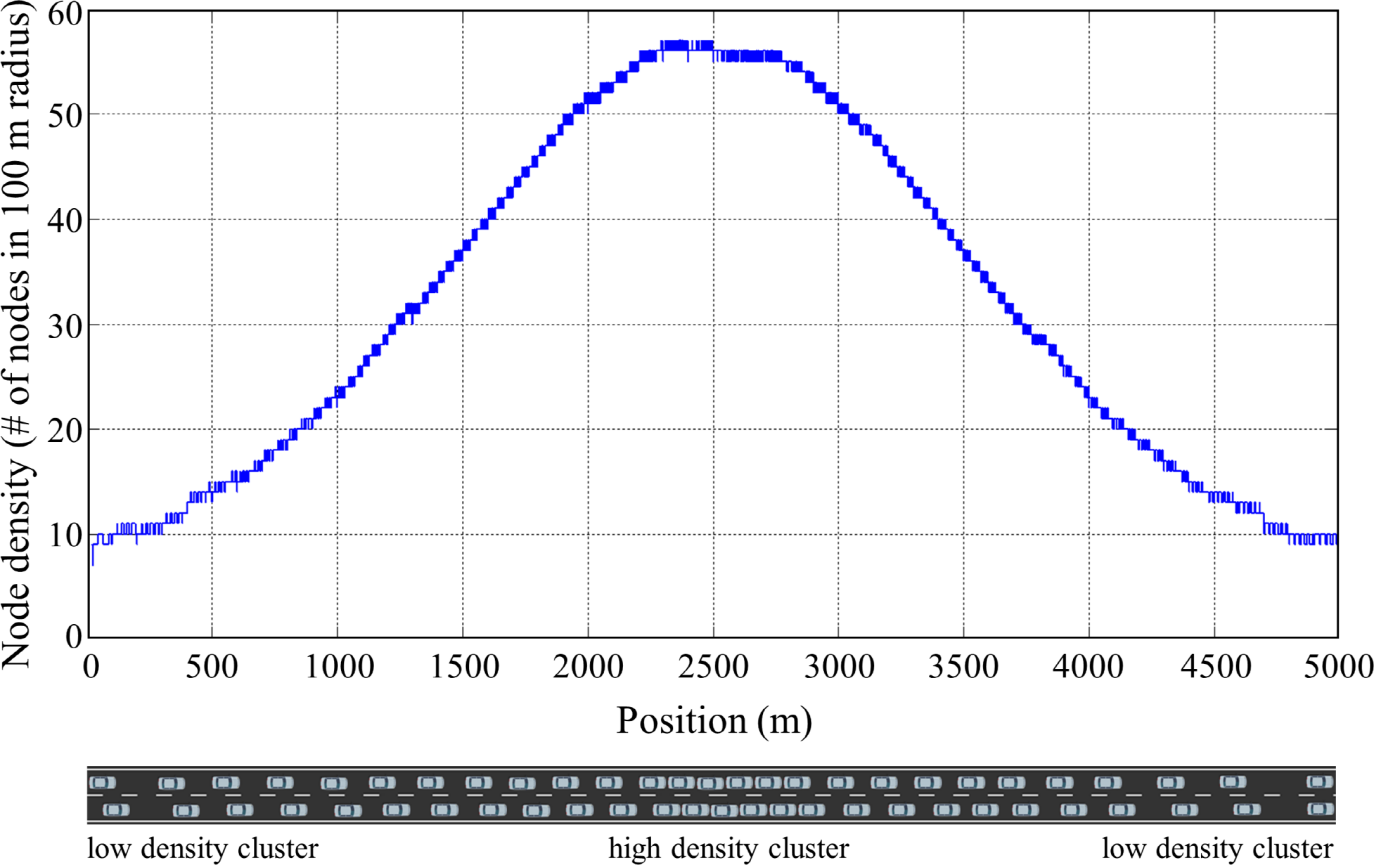
Vehicle distribution of *Scenario 1*. 796 vehicles are positioned on a 5km straight lane with Gaussian distribution (max density = 55 and SD = 1000 m) of vehicle density.

In this simulation scenario, we evaluated the performance of the proposed algorithm in a static traffic situation where vehicles do not move. The static traffic scenario allows us to show fundamental characteristics of the proposed algorithm. A total of 796 vehicles are positioned on a straight double lane road (5 km) to have Gaussian distribution of vehicle density where maximum density and standard deviation (SD) were set to 55 vehicles in 100 m radius and 1000 m, respectively, as shown in Fig 7. Three transmit power control methods including constant transmit power, the Algorithm 1 and the Algorithm 2 were applied for the congestion control. For the Algorithm 1 and 2, initially, the transmit power of all vehicle was set to 11 dBm and differential value *ε* was set to 0.5 dBm. In case of constant transmit power, the transmit power of all vehicle was set to 19 dBm. The simulations were executed for 30s until the simulation converged to steady states, and *CR*, *BL*, and packet Inter-Reception Time (*IRT*) of all vehicles at the end of the simulation were recorded to compare the performance of the algorithm. The *IRT* is the time between two sequential packet receptions for a particular sender-receiver pair, and this matric is used to assess the temporal aspect of awareness in each transmission power control algorithm.

**Fig 8:**
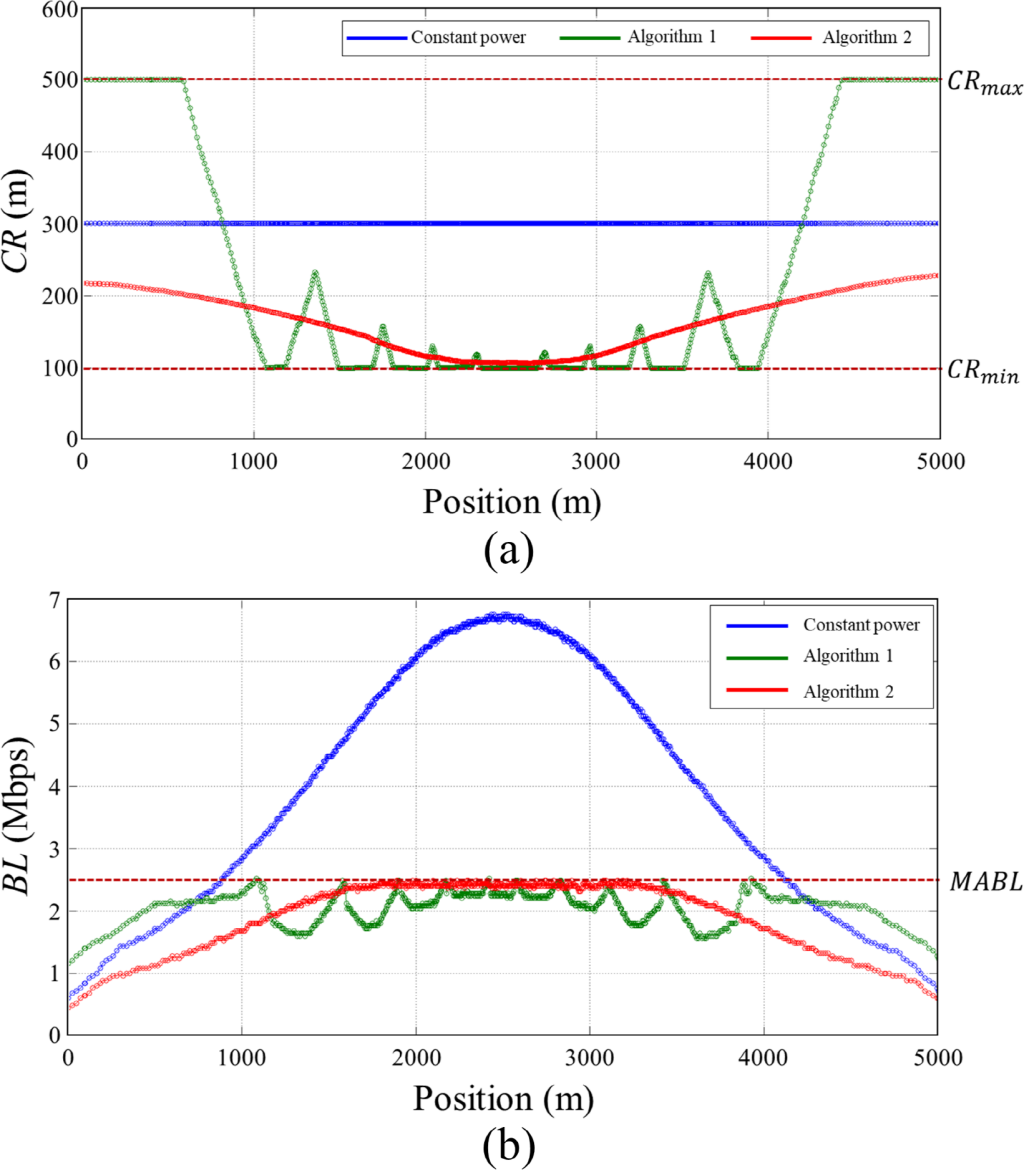
Simulation results of *Scenario 1*. (A) *CR*, (B) *BL*.

Fig 8 shows *CR* and *BL* of the vehicles in the simulation *Scenario 1* along with applied transmit power control methods. In the case with constant transmit power, *BL* varied depending on the vehicle densities, and exceeded *MABL* in a high vehicle density region (800~4200 m). It increased up to 6.7 Mbps which is far beyond *MABL* at the position of the highest vehicle density (2500 m). In this case, *BL* increased as the number of neighbor vehicles increase, and finally the network collapsed due to the packet collision. On the other hand, in the case of Algorithm 1, *BL* of all vehicles was maintained below and close to *MABL* as presented with green line in Fig 8B. It successfully controlled transmit power of each vehicle according to the nearby vehicle densities so that the network resources were efficiently allocated within a given network capacity. However, there were drastic variations in *CR* among nearby vehicles, and this results in an unfair situation in which the sacrifice of particular vehicles occurs for the stability of the entire network. In the case of the Algorithm 2, *CR* varied smoothly according to the vehicle densities and there were no drastic variations in *CR* causing unfairness situation. *BL* also varied smoothly below *MABL*, and converged to *MABL* as the vehicle density increased. In low density regions (0~1200 m and 3800~5000 m), *BL* of the Algorithm 2 is less than that of Algorithm 1 because Algorithm 2 restricts increase of transmission power considering nearby vehicles’ transmission power level to allocate network resources fairly to the vehicles participated in the network. Even though the Algorithm 2 sacrifices little network resources in a low density region compared to the Algorithm 1, transmit power control is done fairly through load sharing with neighboring vehicles without any sacrifice of specific vehicles.

**Fig 9:**
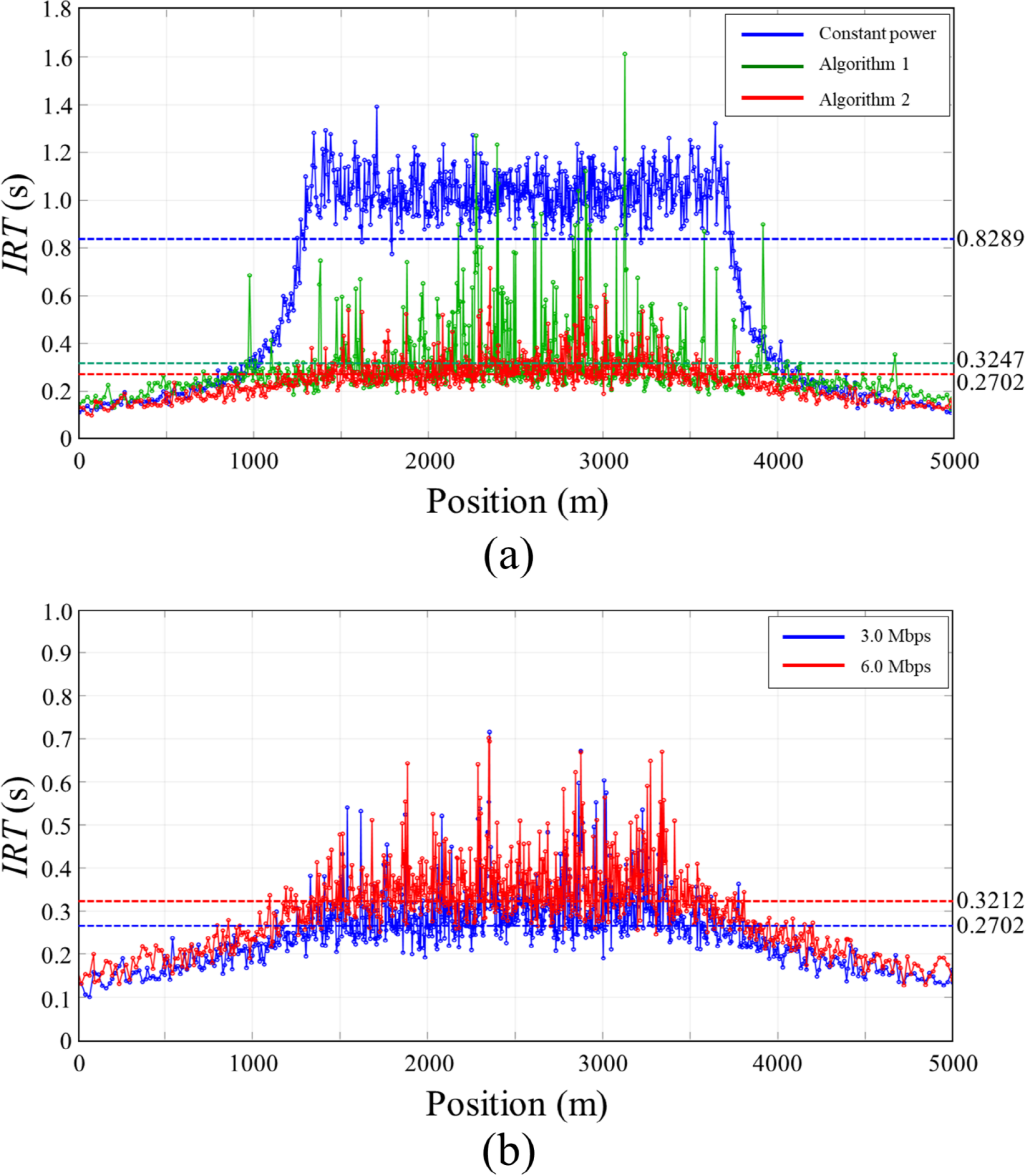
*IRT* measured on *Scenario 1*. (A) *IRT* according to the transmit power control algorithm, (B) *IRT* according to the data rate.

Fig 9A shows the *IRT* of the vehicles along with applied transmit power control methods. The *IRT* increases as the *BL* increases. The higher *BL* leads to the higher packet collisions, and it results in the larger *IRT*. The larger *IRT* mean the older age of information received from its neighboring vehicles. As shown in the figure, the *IRT* of constant transmit power reaches to 1.4 second while the proposed algorithm (Algorithm 2) maintains the *IRT* below 0.4 second. The average value of the *IRT* was 0.8289, 0.3247, and 0.2702 for constant transmit power, Algorithm 1, and Algorithm 2 respectively. This means the proposed algorithm provides more recent information on its neighboring vehicles compared to the other power control algorithm. Fig 9B shows the *IRT* of the proposed algorithm for the data rate of 3 Mbps and 6 Mbps. The simulation scenarios presented in this manuscript uses 3 Mbps data rate, but the DSRC-based safety communications use 6 Mbps data rate. In general, the larger data rate leads to higher packet loss rate and it results in the larger *IRT*. In the *Scenario 1*, the packet loss rate in 6 Mbps data rate was 7%~8% higher than that in 3 Mbps, and it resulted in the increase of *IRT* about 0.05 second in average as shown in Fig 9B. The average *IRT* was 0.2702 and 0.3212 second for 3 Mbps and 6 Mbps data rate respectively.

#### Scenario 2 (dynamic)

**Fig 10:**
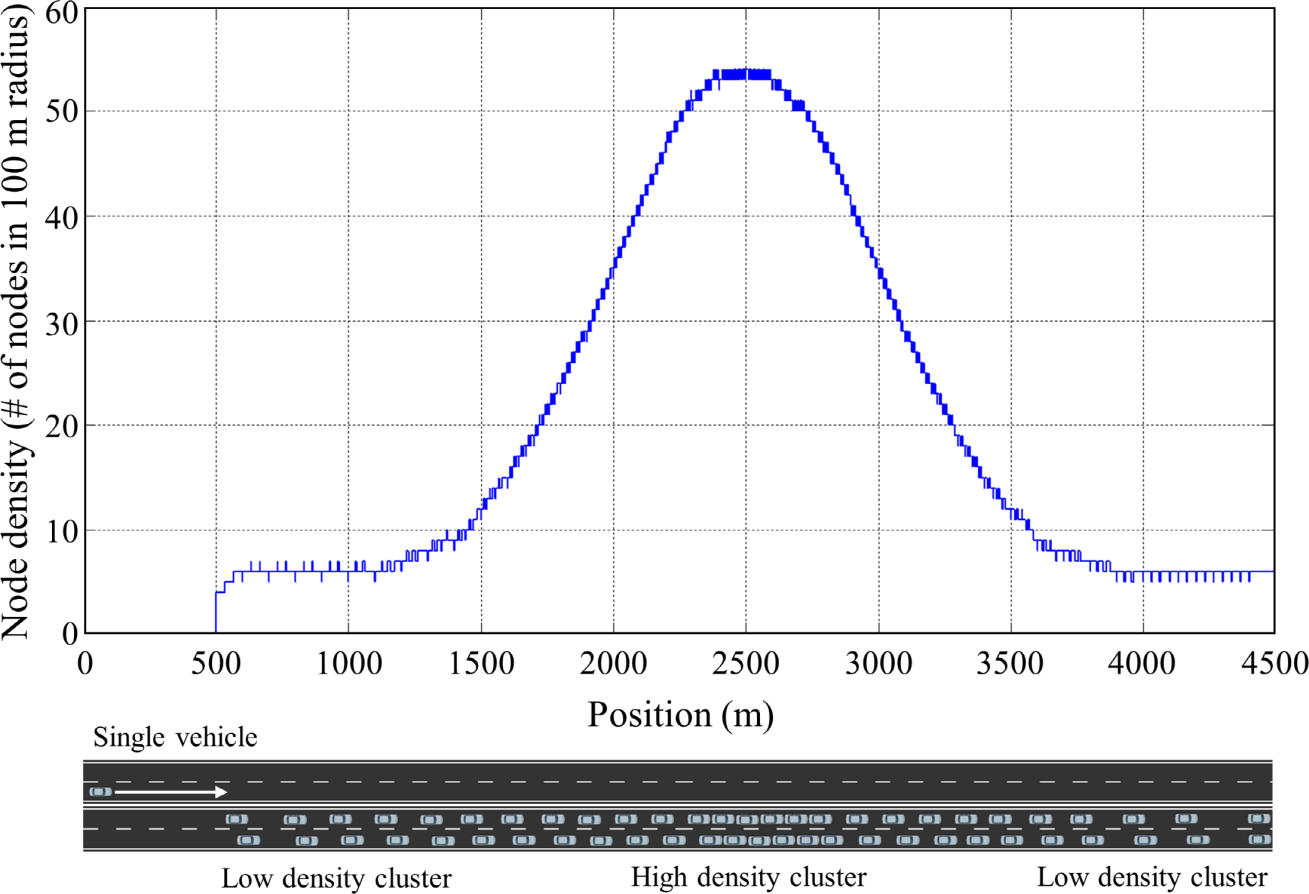
Vehicle distribution of *Scenario 2*. A total of 420 static vehicles are positioned on a 4500 m straight lane from 500 m to 4500 m with Gaussian distribution (max density = 55 and SD = 500 m) of vehicle density. Single vehicle positioned on the other road moves with 10m/s from the left end to the right end of the road.

In this scenario, we evaluated the performance of the proposed algorithm in a time-varying traffic density. Total of 420 static vehicles are positioned on a 4.5 km straight double lane road from 500 m to 4500 m with Gaussian distribution of vehicle densities (maximum density = 55, SD = 500) as shown in Fig 10. Single vehicle is positioned on the other lane at 0 m and moves with 10 m/s toward the static vehicle cluster from the left to the right end of the road. The moving vehicle experiences the varying traffic densities as it passes through the static vehicle cluster. We observed evolution of moving vehicle’s BL, *CR* and *IRT* to test the ability of the proposed algorithm to adopt a time-varying traffic situation. The transmit power levels were set as in *Scenario 1*.

**Fig 11:**
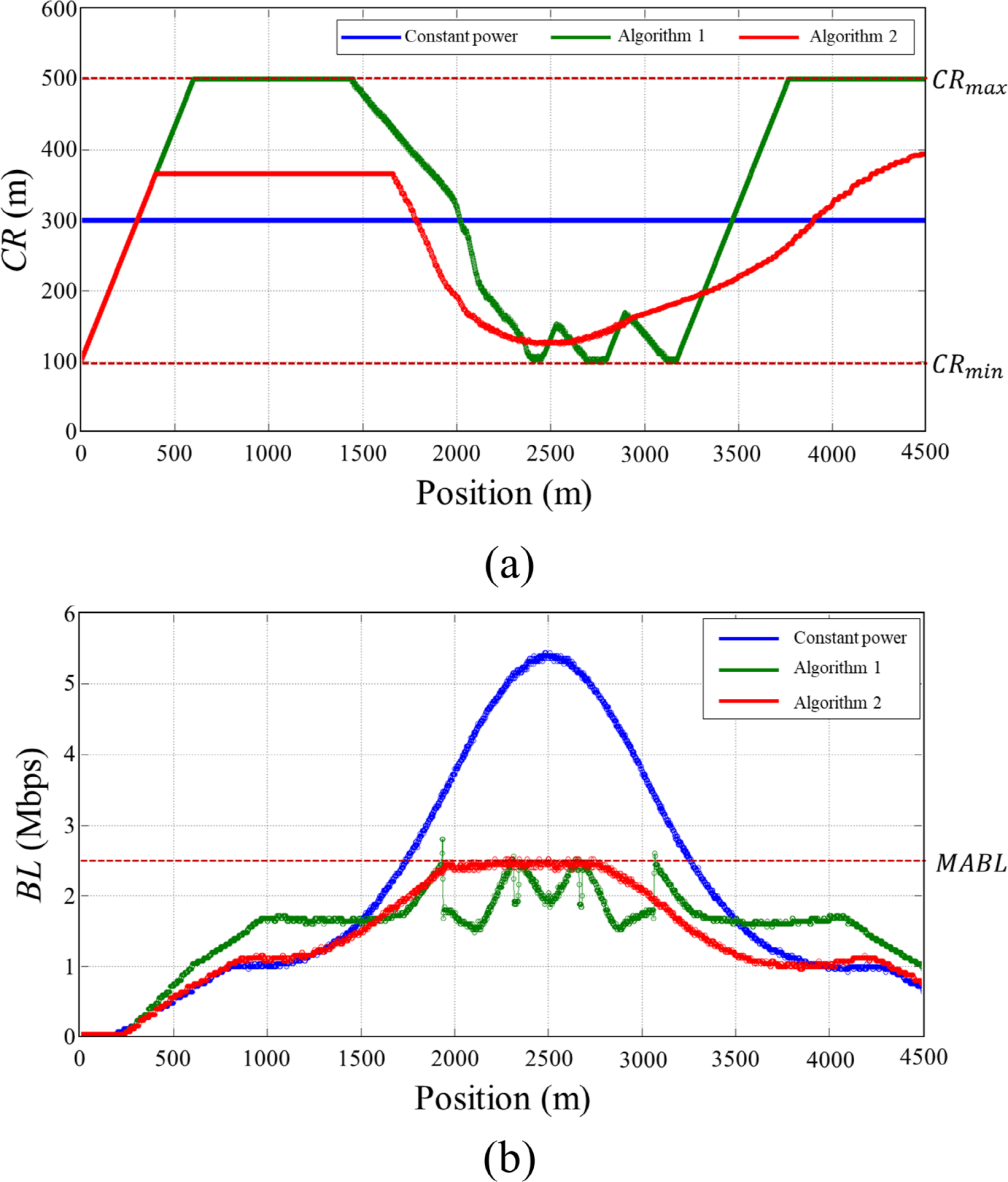
Simulation results of *Scenario 2*. (A) *CR*, (B) *BL*.

Fig 11 shows *BL* and *CR* of the moving vehicle according to the position on the road. In the case of constant power, *BL* of the moving vehicle varies according to the nearby vehicle densities, and it exceeds *MABL* when it passes through the high-density region. In the case of the Algorithm 1, the moving vehicle increases its transmit power rapidly and *CR* reached to the maximum *CR* (500 m) at 600 m position. *CR* begins to decrease as the moving vehicle entered to the high-density region until it reached to the minimum *CR* (100 m). In the region between 2400 m and 3200 m, *CR* drastically fluctuated even though the nearby traffic density of the moving vehicle varies gradually with Gaussian distribution because of unfairness problem of the Algorithm 1. *BL* of the moving vehicle drastically changed from 1.5 Mbps to 2.5 Mbps in the region between 1900 m and 3100 m because there were drastic changes in *CR* of static vehicle cluster. *BL* is controlled bellow *MABL* even the vehicle go through the high density region, but the fairness problem occurred as in the static situation. In the case of the Algorithm 2, *CR* varies more slowly and smoothly than that of the Algorithm 1. *BL* gradually increased as the vehicle density increases and converged to *MABL* at 1900 m. And then it gradually decreased as the vehicle density decreases from 2700 m as shown in Fig 11B. Note that there were no drastic changes in both *CR* and *BL* of the moving vehicle, and also *BL* was adaptively adjusted within a given network capacity *MABL* for the entire simulation.

**Fig 12:**
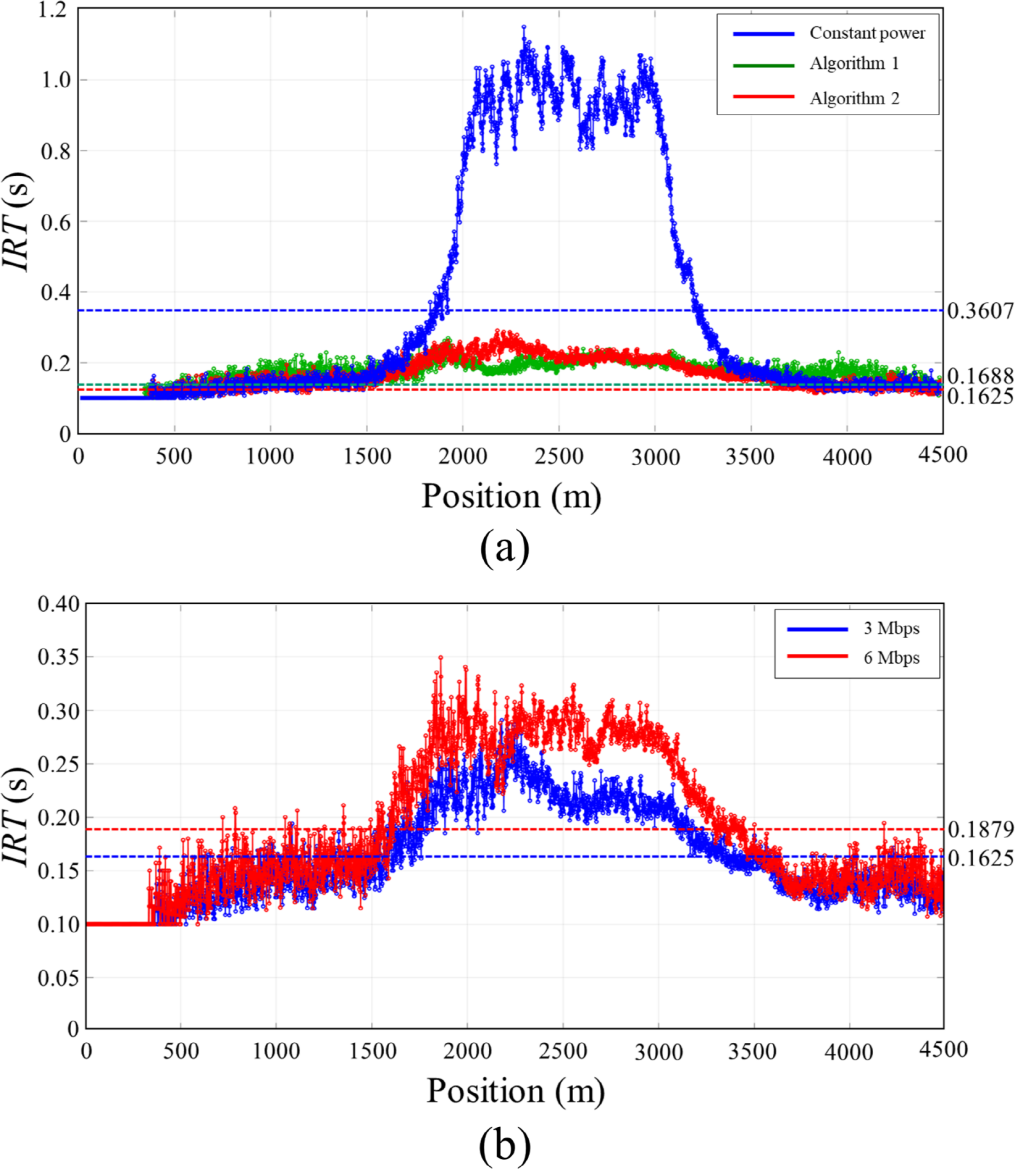
*IRT* measured on *Scenario 2*. (A) *IRT* according to the transmit power control algorithm, (B) *IRT* according to the data rate.

Fig 12A shows the *IRT* of the vehicles along with applied transmit power control methods measured in *Scenario 2*. The *IRT* of the Algorithm 2 presented the minimum value among the three transmit power control algorithm. The average value of the *IRT* was 0.3607, 0.1688, and 0.1625 for constant transmit power, Algorithm 1, and Algorithm 2 respectively. Similar with the results shown in *Scenario 1*, the proposed algorithm provides the most recent information on its neighboring vehicles compared to the other power control algorithm. Fig 12B shows the *IRT* of the proposed algorithm for the data rate of 3 Mbps and 6 Mbps measured in *Scenario 2*. The average value of *IRT* was 0.1625 and 0.1688 second for 3 Mbps and 6 Mbps data rate respectively. Due to the higher packet collision in 6 Mbps data rate, the data received in the vehicle was slightly delayed compared to that in 3 Mbps data rate.

**Fig 13:**
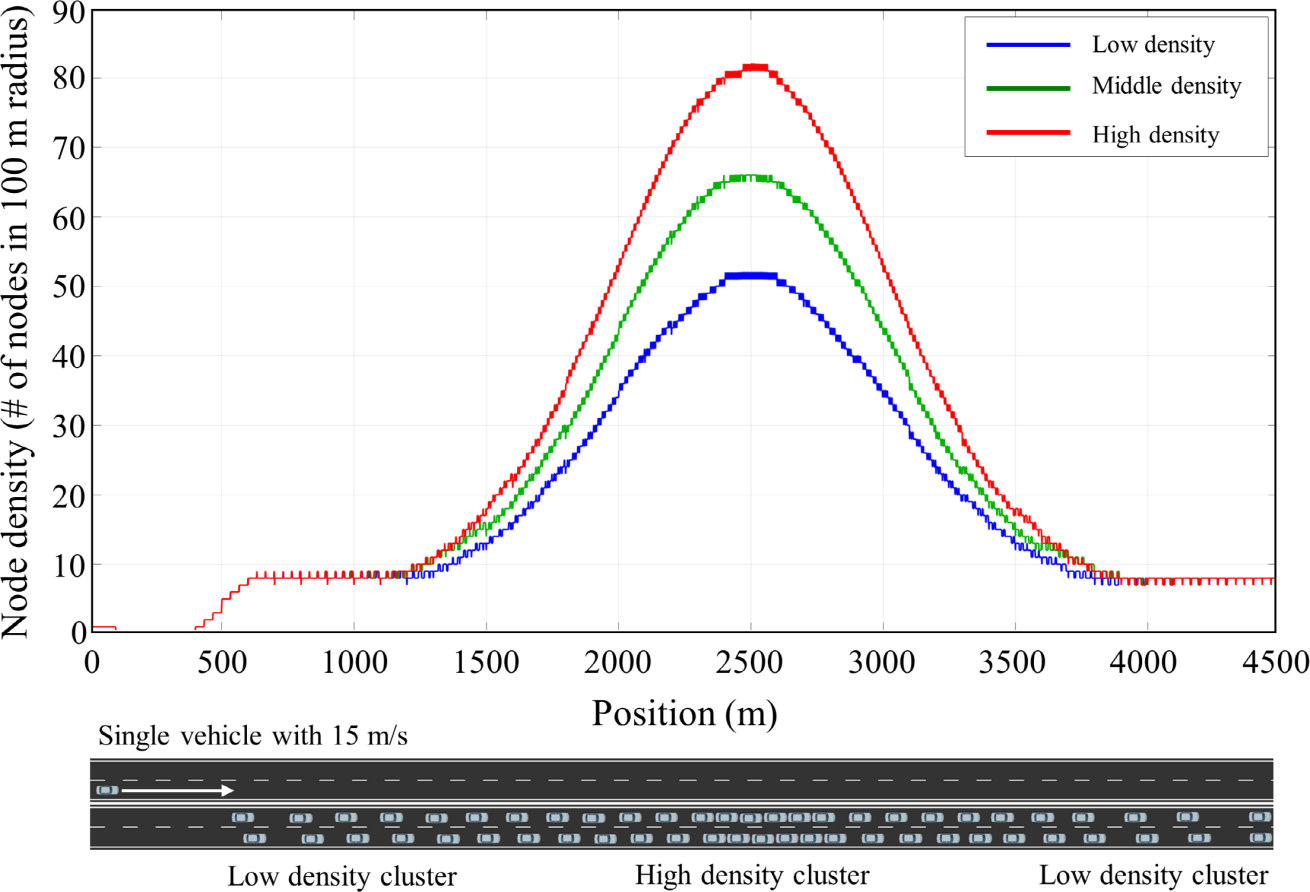
Vehicle distribution of *Scenario 2* with three vehicle densities (low, middle, and high density).

To further explore the impact of traffic flow on the communication performance of the proposed algorithm, we compared the performance with various vehicle densities as shown in Fig 13. The maximum density of the static vehicles in Gaussian distribution was set to 50, 65 and 80 (referred to as low, middle, and high vehicle densities respectively). For each vehicle density, the *BL* and *IRT* was measured and compared with each other.

**Fig 14:**
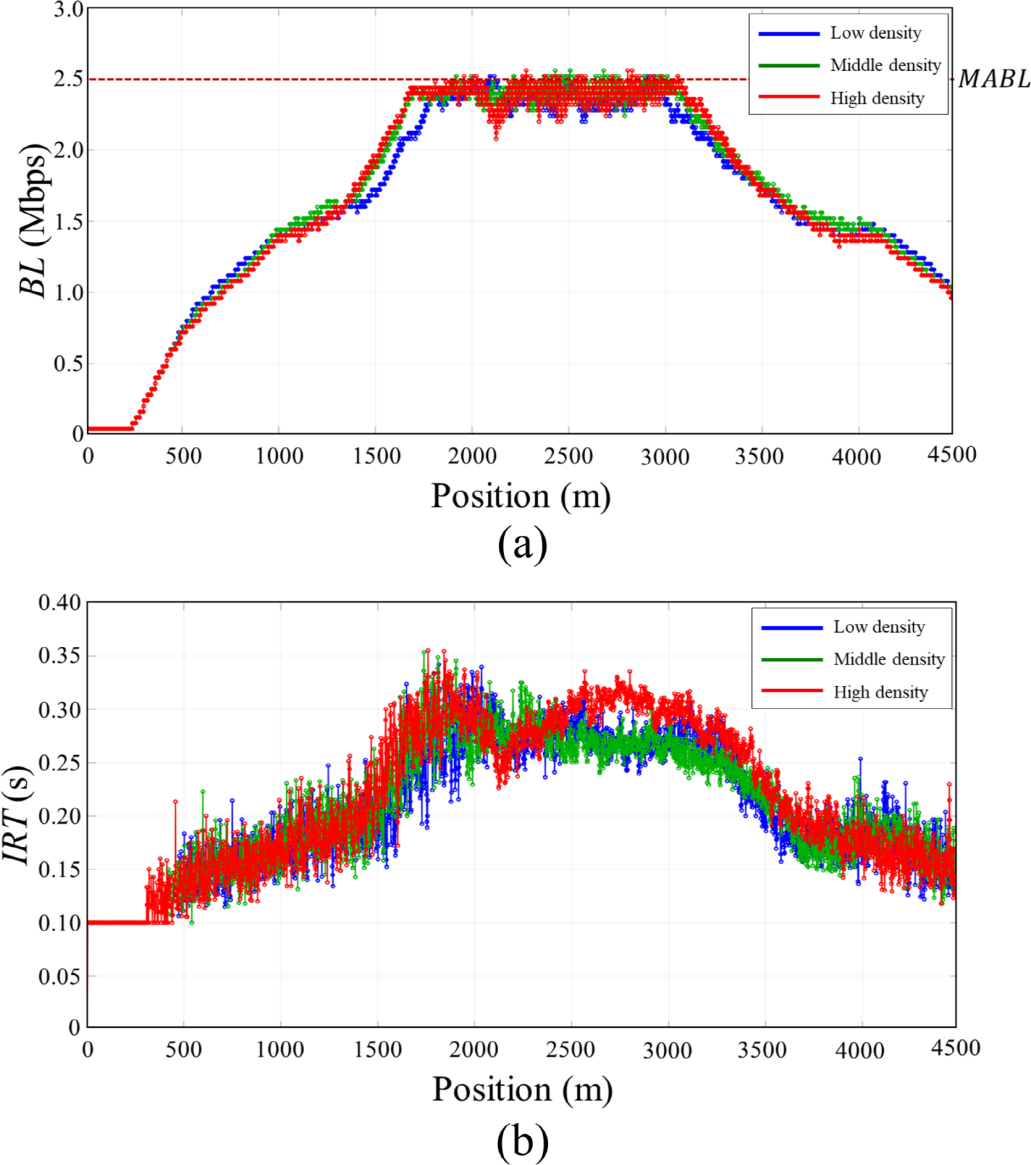
Communication performance according to various density of the static vehicles. (A) *BL* (B) *IRT*.

Fig 14 shows the *BL* and *IRT* of the proposed algorithm according to the traffic densities. In this simulation, the speed of moving vehicle was set to 15 m/s. The moving vehicle experienced the varying vehicle densities as it goes through the static vehicle clusters and adaptively adjusted its transmit power to maintain the *BL* under *MABL*. As shown in Fig 14A, the *BL* of the moving vehicle was successfully managed under *MABL* for all vehicle densities. The average value of the IRT was 0.2036, 0.2054, and 0.2140 second for low, middle, and high vehicle density respectively. The *IRT* was managed with a similar level even though the vehicle density increases as the proposed algorithm adaptively controls the *BL* according to the vehicle density.

#### Scenario 3 (dynamic)

**Fig 15:**
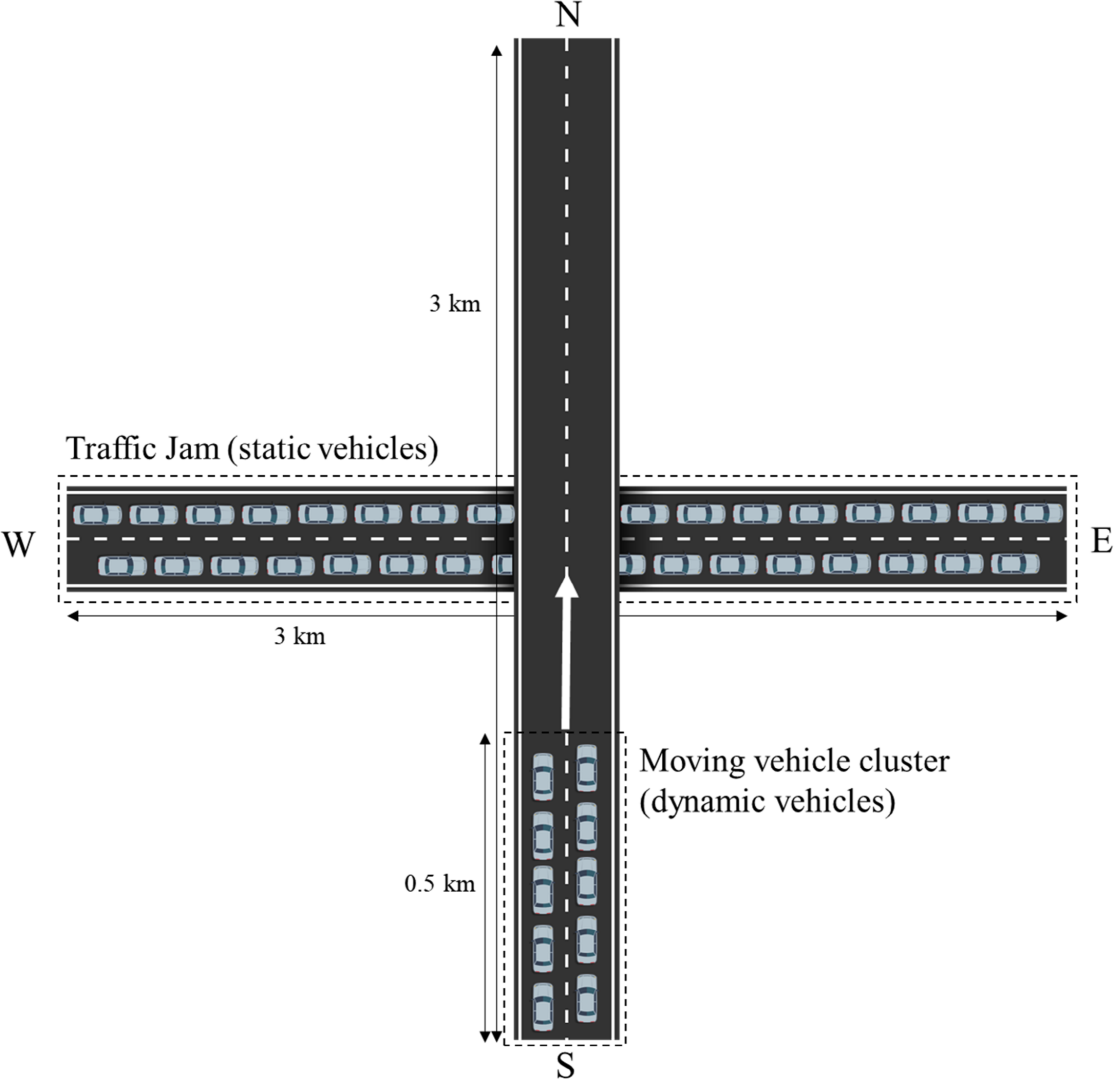
Configuration of vehicles in simulation Scenario 3. A total of 300 static vehicles are positioned on a straight double lane highway (3000m) oriented in west-east. A set of 50 moving vehicles positioned on the south-west highway (0~500 m) moves from south to north toward the static cluster with 20 m/s.

In this scenario, we examined the proposed algorithm in a demanding situation. A static cluster of 300 vehicles mimicking traffic jam situation in a highway were positioned along a 3000 m straight double lane road segment oriented west-east, and it is intersected at the middle (1500 m) by another highway overpassing it in south-north direction as shown in Fig 15. A set of 50 moving vehicles positioned on the south-west highway segment (0~500 m) moves from south to north toward the static cluster with 20 m/s. In this dynamic scenario, we tested how the proposed algorithm adapts demanding variations of traffics caused by moving vehicle cluster. The simulation was executed for 160 s until the moving vehicle cluster completely get out from the effect of static vehicle cluster, and we observed *CR* and *BL* of all static vehicles during entire simulation.

**Fig 16:**
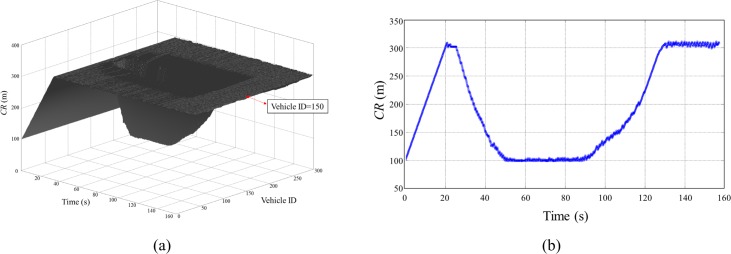
Simulation results (*CR*) of *Scenario 3*. (A) *CR* of all vehicles according to the simulation time, (B) *CR* of the vehicle (ID = 150) according to the simulation time.

**Fig 17:**
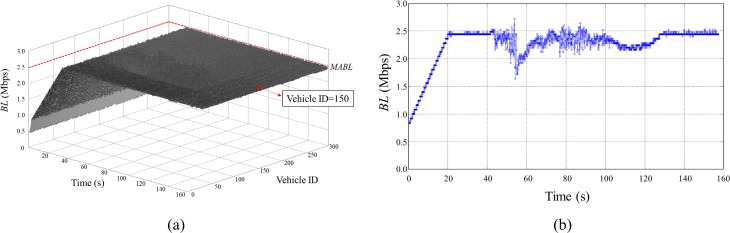
Simulation results (*BL*) of *Scenario 3*. (A) *BL* of all vehicles according to the simulation time, (B) *BL* of the vehicle (ID = 150) according to the simulation time.

Figs 16 and 17 shows time evolution of *CR* and *BL* of all stationary vehicles during the entire simulation of the Algorithm 2. The *CR* and *BL* of a vehicle at the center of the cluster (vehicle ID = 150) are separately presented to show the value clearly (Fig 16B and Fig 17B). At the beginning of the simulation, all vehicles increased *CR* until *BL* reached to *MABL* (20 s). When the moving vehicle cluster entered to the *CR* of static cluster (25 s), then the vehicles positioned at the middle of the static cluster began to decrease their *CR* to maintain *BL* below to *MABL* as shown in Figs 16 and 17. As more vehicles flowed into the influence range of the static cluster, *CR* of the vehicles at the middle of the static cluster reached to *CR*_*min*_. A little fluctuation of the *BL* occurs under *MABL* in a highly crowed and dynamics traffic situations up to the time when the moving vehicle cluster completely goes out (43~128 s), and thereafter converged to *MABL* again. The simulation results show that the Algorithm 2 fairly allocates network resources without causing communication congestion in the demanding traffic situation. As a result, in terms of resource sharing, all vehicles were kept at a similar level compared to the surrounding vehicles, which means that all the vehicles maintained the safety state almost the same as the surrounding vehicles.

## Conclusions

This paper proposes a distributed transmission power control algorithm in order to overcome existing challenges in VANET communications, and to support active applications for improving road safety. Transmission power control has been identified as a key parameter for the mitigation of channel congestion caused by the transmission of beacon messages. Therefore, a mechanism to control the *BL* of the channel was needed. High load on the channel results in a large amount of packet collisions, which decreases the probability of successful receipt of beacon messages, resulting in a lower safety level for the roadway.

To address these issues in VANETs, the authors propose a distributed transmission power adjustment algorithm for communication congestion control and vehicle awareness enhancement. Distributed strategies that control a vehicle’s communication behavior in a cooperative manner were utilized to keep the *BL* below a predefined threshold. The performance of the proposed algorithm was evaluated in various traffic situations using a network and mobility simulation model. The simulation results show that:

- The proposed algorithm controls communication congestion on the basis of *BL* restriction. A fully distributed strategy was implemented, capable of controlling the transmission power of each vehicle, such that the channel load in a wireless network is kept under a given threshold. A distributed power control algorithm is proposed accordingly, to achieve the maximum utilization of beaconing channel capacity without violating the assigned emergency channel load. To allow space for event-driven messages, beaconing messages are restricted below the *MABL*. Simulation results show that the proposed algorithm is successfully able to control the beaconing load on the channel while still ensuring that the probability of beacon reception is high within the “safety distance” of the sending vehicle.
- The proposed algorithm enhances a vehicle’s awareness of its surroundings by using a distributed approach. Communications congestion was identified as the main challenge for beacon messages, stressing the need for a strategy to carefully control the quantity and distribution of beacon messages. A high beaconing load on the communications channel is likely to result in an increase in packet collisions, and consequently, a decrease in roadway “safety level.” This aspect is shown with the *IRT* value which is the matric used to assess the temporal aspect of the awareness. The *IRT* of the proposed algorithm was the minimum among the other control algorithm, and these results prove that the proposed algorithm provides the most recent information on its neighboring vehicles. Beacon messages are most relevant at close distances from the sender, becoming less relevant at further distances, according to the “safety distance” of each vehicle. Good awareness was identified as a required criterion for communication protocols in order to achieve safety, as it is clear that if a vehicle is not able to periodically collect its neighbors’ information, it can be dangerously unaware of the situation on the roadway. Simulation results show that the proposed transmission power control algorithm successfully maximizes the situational awareness of each vehicle.
- The proposed algorithm controls the transmission power without sacrificing specific vehicles awareness by sharing resources fairly with nearby vehicles. In a congested situation, a particular vehicle may be at risk if it acquires less peripheral information than the surrounding vehicle. In other words, if a vehicle has a transmission power that is too small compared to the surrounding, then the surrounding vehicle may not be able to obtain information about the vehicle. In this paper, we solve this problem by considering average power level of neighboring vehicles when increasing the transmit power. The simulation results show that the proposed power control algorithm maintains proper balance in terms of fairness and efficiency.

By controlling the transmission power, the *BL* on a distressed node can be reduced, and all nodes are able to obtain sufficient information to provide situational awareness by individually adjusting their transmission power.

## Supporting information

S1 File Simulation result data of three traffic scenarios. (ZIP)

S2 File Executable files of the simulation of traffic scenarios presented in the paper. (ZIP)

S1 Movie Demonstration of the congestion control in the traffic scenarios presented in the paper. (MP4)

## References

[1] Traffic Safety Facts Research Note, National Highway Traffic Safety Administration [Internet]. Crashstats.nhtsa.dot.gov. 2017. Available from: https://crashstats.nhtsa.dot.gov/Api/Public/ViewPublication/812318

[2] WHO | World report on road traffic injury prevention [Internet]. Who.int. 2017. Available from:http://www.who.int/violence_injury_prevention/publications/road_traffic/world_report/en/

[3] IEEE Standard for Wireless Access in Vehicular Environments (WAVE)—Networking Services, IEEE Std. 1609.3–2010. Available from: https://standards.ieee.org/findstds/standard/1609.3-2016.html

[4] SAE International, Dedicated Short Range Communications (DSRC) Message Set Dictionary, SAE J2735, Nov. 2009. Available from: https://saemobilus.sae.org/content/j2735_200911

[5] SAE International, On-Board System Requirements for V2V Safety Communications, SAE J2945/1 Mar. 2016. Available from: https://saemobilus.sae.org/content/j2945/1_201603

[6] ETSI TS 102 687, Intelligent Transport Systems (ITS); Decentralized Congestion Control Mechanisms for Intelligent Transport Systems operating in the 5 GHz range; Access layer part July 2011. Available from: http://www.etsi.org/deliver/etsi_ts/102600_102699/102687/01.01.01_60/ts_102687v010101p.pdf.

[7] Eenennaam M, Wolterrink W, Karagiannis G and Heijenk G. Exploring the solution space of beaconing in VANETs, Proceedings of the 1st IEEE Vehicular Networking Conference–VNC09. 2009.

[8] Sommer C, Tonguz O and Dressler F. Traffic Information Systems: Efficient Message Dissemination via Adaptive Beaconing, IEEE Communication Magazine. 2011; 49(5): 173–179.

[9] Kim B, Kang I, Kim H. Resolving the Unfairness of Distributed Rate Control in the IEEE WAVE Safety Messaging. IEEE Transactions on Vehicular Technology. 2014; 63(5): 2284–2297.

[10] Egea-Lopez E, Pavon-Mariño P, Distributed and Fair Beaconing Rate Adaptation for Congestion Control in Vehicular Networks, IEEE Transactions on Mobile Computing. 2016; 15(12): 3028–3041

[11] Egea-Lopez E, Pavon-Mariño P, Fair Congestion in Vehicular Networks With Beaconing Rate Adaptation at Multiple Transmit Powers, IEEE Transactions on Vehicular Technology. 2016; 65(6): 3888–3903

[12] Artimy MM, Robertson W, Phillips W. Assignment of dynamic transmission range based on estimation of vehicle density. Proceedings of the 2nd ACM international workshop on Vehicular ad hoc networks—VANET '05. 2005.

[13] Artimy MM. Local Density Estimation and Dynamic Transmission-Range Assignment in Vehicular *Ad Hoc* Networks. IEEE Transactions on Intelligent Transportation Systems. 2007;8(3):400–412.

[14] Torrent-Moreno M, Santi P, Hartenstein H. Fair sharing of bandwidth in VANETs. Proceedings of the 2nd ACM international workshop on Vehicular ad hoc networks—VANET '05. 2005.

[15] Torrent-Moreno M, Santi P, Hartenstein H. Distributed Fair Transmit Power Adjustment for Vehicular Ad Hoc Networks. 2006 3rd Annual IEEE Communications Society on Sensor and Ad Hoc Communications and Networks. 2006.

[16] Torrent-Moreno M, Mittag J, Santi P, Hartenstein H. Vehicle-to-Vehicle Communication: Fair Transmit Power Control for Safety-Critical Information. IEEE Transactions on Vehicular Technology. 2009;58(7):3684–3703.

[17] Mittag J, Schmidt-Eisenlohr F, Killat M, Härri J, Hartenstein H. Analysis and design of effective and low-overhead transmission power control for VANETs. Proceedings of the fifth ACM international workshop on VehiculAr Inter-NETworking—VANET '08. 2008;.

[18] Lu H, Poellabauer C. Balancing broadcast reliability and transmission range in VANETs. 2010 IEEE Vehicular Networking Conference. 2010.

[19] Chuang C, Kao S. A probabilistic discard congestion control for safety information in vehicle-to-infrastructure vehicular network. The 40th International Conference on Computers & Indutrial Engineering. 2010.

[20] Mo Y, Yu D, Song J, Zheng K, Guo Y, A Beacon Transmission Power Control Algorithm Based on Wireless Channel Load Forecasting in VANETs, PLOS ONE. 2015 10(11): e0142775 10.1371/journal.pone.0142775 26571042 PMC4646483

[21] Huang CL, Sengupta R, Krishnan H, Fallah YP. Implementation and evaluation of scalable Vehicle-to-Vehicle transmission control protocol. IEEE Communications Magazine. 2011; 49(11).

[22] Baldessari R, Scanferla D, Le L, Zhang W, Festag A, Joining forces for vanets: A combined transmit power and rate control algorithm, Proceeding of the 6th International Workshop on Intelligent Transportation (WIT), 2010.

[23] Tielert T, Jiang D, Hartenstein H, Delgrossi L, Joing Power/Rate Congestion Control Optimizing Packet Reception in Vehicle Safety Communications, Proceedings of the 10th International Workshop on Vehicular Inter-Networking, Systems, and applications. 2013.

